# Supplementary material for: Evolution of Medical Students’ Perception of the Patient’s Right to Privacy
Source: Int J Environ Res Public Health. 2022 Sep 4;19(17):11067. doi: 10.3390/ijerph191711067 (PMC9517786; doi:10.3390/ijerph191711067)
Supplement: Supplementary file 1 [file ijerph-19-11067-s001.zip › ijerph-1852573-supplementary.pdf]

SUPPLEMENTARY MATERIAL

Supplementary Table S1. Questions related to personal information of the students.

**Questions related to personal information**
1. What is your date of birth?<br>2. What is your gender?<br>3. What is your province of habitual residence (non-academic period)?<br>4. Who do you live with during the academic year?<br>5. What level of education has your father reached?<br>6. What level of education has your mother reached?<br>7. In which year did you begin your degree in Medicine?<br>8. What is your province of habitual residence (academic period)?<br>9. Which country do you live in?<br>10. In which Medical School do you study?<br>11. Which is your current year of study?<br>12. Do you have subjects pending from previous years?<br>13. If yes, how many?<br>14. What is your current average grade?<br>15. In which medical or surgical services have you rotated?<br>16. In which health-care facilities have you rotated?

Supplementary Table S2. Articles related to patients' right to privacy from the SSI/81/2017 Spanish Order.

Article	Contents
Article 4	Patient's right to a decent treatment and supervised action of the personnel in training.
Article 4.1	In health-care processes, people have the right to receive decent and respectful treatment at all times and in all circumstances. All persons (students, health and non-health professionals) who participate in any way in a healthcare process must respect the privacy and dignity of patients, adopting attitudes that conform to generally accepted standards of courtesy and kindness and using appropriate language.
Article 4.2	It will be promoted that the personnel in training have a behavior that corresponds to the ethical contents of the official programs of the specialty or, where appropriate, study plans and the deontological codes of the different health professions, as well as to know the operation of the Healthcare Ethics/Research Committees, so that personnel in training acquire skills for making decisions guided by ethical values.
Article 4.3	Resident doctors and students in training, who carry out rotations or practice in health centers, will respectively have a specialized training tutor or a clinical tutor, a professor with a related position, or an associate professor of health sciences, belonging to the staff of the health center where clinical practices are developed. The aforementioned teaching managers will be the reference persons to whom other professionals of the center or people from outside the same may contact, in relation to the activities carried out by residents and students in training.
Article 4.4	Personnel in training of any year will act subject to the indications of their teaching managers/tutors and in their absence they will submit, in any case, to the indications of the specialists of the healthcare unit, without prejudice to, once concluded the care process, inform, ask, even question, within the rules of healthy criticism, decisions and other aspects of care practice.
Article 4.5	The health-care center will encourage, to the extent possible, the use of standardized/simulated patients/mannequins or other simulation techniques for clinical situations, so that personnel in training can acquire clinical skills and technical and teamwork skills, prior to real and necessary contact with the patient.<br>In the collaboration agreements signed with the educational centers, it will be foreseen that they will also carry out simulated activities in their own centers prior to the start of practices at the health-care center.
Article 4.6	It is responsibility of the management of the health-care centers to ensure the full integration of students and residents in training in the care dynamics of the center, and to ensure the assumption and compliance with the regulations governing its functioning, especially those related to its uniformity and identification, with compliance with the ethical principles and basic rules of action and with the specific guidelines contained in this protocol.
Article 4.7	Students and resident doctors will have the duty to integrate into the care dynamics of the center subject to the regulations that govern its operation, especially those related to its uniformity and identification, compliance with ethical principles and basic rules of action and guidelines specific contained in this protocol.
Article 4.8	Health-care institutions must inform students and residents of specialties in health sciences about personal data protection measures when using electronic devices (laptops, USB sticks, etc.). In no case may this information be shared using information systems and/or computer tools/social networks, which are not subject to the security systems of health centers.
Article 5	Guidelines for action in relation to the presence of STUDENTS of health sciences degrees in healthcare processes.

Article 5.1	The next will be considered as students:<br><br>a) University students of qualifications that enable the exercise of health professions titled and regulated in Health Sciences: Medicine, Pharmacy, Dentistry, Nursing, Physiotherapy, Occupational Therapy, Podiatry, Optics-optometry, Speech Therapy, Nutritionist Dietitians, General Health Psychology.<br>b) University students of Master, Doctorate, titles related to health professions.<br>c) Professional-formation students from the healthcare family, both from mid-level technicians and higher-level technicians.<br>d) Students from other university degrees or professional training with internships in health centers.
Article 5.2	Patients have the right to know that there are students present in their healthcare process. The management of the health-care center (and not the university, school or training center of origin) will provide them with an identification card that will be placed in a visible place of the uniform containing the personal data, photograph and express reference to the group to which the student belongs among the aforementioned in point 5.1, in order to facilitate its recognition by users and professionals of the center.<br>At the end of the training period at the center, it will be mandatory to return the identification card to the center's personnel services.
Article 5.3	Prior to the start of the care process, the professional responsible for it (unit specialist, tutor or resident doctor authorized by their tutor) will inform the patient or their representative about the presence of students, requesting their verbal consent to witness the clinical process.<br>In accordance with the provisions of Article 7.3 of the LOPD in relation to Article 8.1 of the LBAP, in the event that the patient refuses, the personnel in training will not be present in the healthcare process.<br>If the patient consents that the students are present during the clinical act, his consent will be reiterated in the event that it is considered appropriate to carry out some type of physical examination, clinical procedure or intervention for training purposes.<br>Notwithstanding the foregoing, it will also be possible to request the patient's global consent for a limited period of time that will be graduated based on the expected time of stay in the health center. Said period may not exceed 15 days.<br>The presence of students may be limited and in particular when the corresponding health professional understands the presence of the student inadequate due to the clinical, emotional or social situation of the patient.<br>To consent to the presence of students and the performance of explorations, interventions or clinical procedures by them, consent may be granted by representation, through the people related to the patient for family or factual reasons or by legal representatives, in the following assumptions provided in Article 9.3 of the LBAP:<br><br>a) When the patient is unable to make decisions, at the judgement of the attending physician, or his physical or mental state does not allow him to take charge of his situation.<br>b) When the patient has the capacity modified judicially and so is stated in court sentence.<br>c) When the under-18 patient is not intellectually or emotionally capable of understanding the scope of the intervention. In this case, consent will be given by the legal representative of the minor, after having heard their opinion, in accordance with the provisions of Article 9 of Organic Law 1/1996, of January 15, on the Legal Protection of Minor Child.<br>In the case of emancipated minors or persons over 16 years of age who are not in cases b) and c) of the previous section, consent cannot be given by representation.

Article 5.4	Students will be supervised at all times, not being able to access the patient or clinical information about it without the direct supervision of the staff of the healthcare center who is responsible for their training in accordance with the provisions of Article 104 of the LGS in relation to the provisions of Royal Decree 1558/1986, of June 28, which establishes the general bases for agreements between universities and health institutions, as well as the provisions of agreements and other regional regulations that in each case may be applicable.
Article 5.5	Except in especial cases consented by the patient, during the clinical acts that are carried out in the presence of the same, no more than three students per patient should be present without prejudice to the participation of others, through the consensual use of screens in another room. Likewise, the repetition sequence with different groups will be planned in reasonable times so that it is not bothersome or intimidating for the patient.<br>The maximum number of people in formation present in presence of the patient may not exceed five, computing the number of residents mentioned in Article 6.5.
Article 7	Guarantee of access to clinical data
Article 7.2	Access to the clinical history of the health service for epidemiological, public health, research or teaching purposes.
Article 7.2.1	The LBAP on its Article 16.3 establishes that access to the medical history for judicial, epidemiological, public health, research or teaching purposes requires the preservation of the patient's personal identification data, separate from those of a clinical healthcare nature. In this way, as a general rule, anonymity is assured, unless the patient himself has given his consent not to separate them.<br>The dissociation of data obliges us to separate the data of scientific utility (in our case, clinical health-care data) from those others that allow us to identify its owner (number of medical history, Social Security, ID number, etc.). The dissociation of data must be carried out by a health professional subject to professional secrecy or another person subject to an equivalent obligation of secrecy.<br>In the field of teaching, students will be able to access the medical record with dissociated personal data or simulated medical records by the person in charge of teaching in order to guarantee that the learning derived from them is carried out respecting the privacy and confidentiality of the health data.
Article 7.4	Access to the SNS Digital Health Record (HCDSNS in Spanish).
Article 7.4.2	Access must be for exclusively healthcare purposes, and only healthcare professionals with healthcare functions who have been previously authorized by their health service can access the system.
Article 7.4.3	Therefore, students of degrees related to health sciences cannot access.
Article 7.5	Copies. Without prejudice to punctual access to the medical record as provided in sections 7.1.1 and 7.1.3, training staff, whether resident doctor or student, may not make copies of the information contained in the medical record by any means and in no format, except with the written consent of the patient or in case of anonymized medical records with the express authorization of the person responsible for the registration of medical records at the proposal of his tutor/teacher.
Article 8	Patients’ right to confidentiality of their health data.
Article 8.1	Article 7.1 of the LBAP establishes that "everyone has the right to have the confidentiality of their health data assured and that no one may access them without prior authorization under the Law".

	This right translates into the duty of confidentiality/secrecy provided for in Article 2.7 of the aforementioned law which establishes that "the person who prepares or has access to the information and clinical documentation is obliged to keep the proper secret."
Article 8.2	Both resident doctors and students are subject to the duty of confidentiality/secrecy, not only during their stay at the healthcare center where they are being trained, but also after their completion, without this duty being extinguished by the death of the patient.<br>The duty of confidentiality affects not only "intimate data" (including psychological data related to ideas, values, beliefs, personal experiences ...) but also biographical data of the patient and his environment (whether intimate or not) whose knowledge by third parties may affect the rights of the person subject to treatment.<br>The duty of confidentiality/secrecy not only refers to the data contained in the patient's medical record but also to those that have been accessed through verbal communication, recordings, videos, as well as the contents in any type of computer, electronic file, telematic or public or private registry, including those related to the degree of disability and genetic information.<br>The duty of secrecy is understood without prejudice to the legal situations in which its maintenance involves risk to the life of the person affected or of third parties or prejudice to Public Health, in which case the managers of the corresponding service/unit will be informed to act accordingly.
Article 8.3	To this end, personnel in training will sign a confidentiality commitment at the beginning of their stay at the health center where they are being trained, that will appear in the Register Book for research personnel and in the student system referred to in Section 9, and in the case of training specialists, in their personal files that the Teaching Commission guards.

Supplementary Table S3. Questions 1-24 regarding students' experience and articles related from the SSI/81/2017 Spanish Order.

Question	Statement	Article
1	During my clinical practice, I am constantly supervised and accompanied by my doctor.	5.4
2	At the beginning of my medical rotations, I have signed a confidentiality commitment provided by the health center where I carry out the practice.	8.3
3*	During my clinical practice, the doctor provides me his/her username and password for the hospital's internal network.	7.4
4	When I am at the hospital, I wear the identification card (name and surname, photograph and "student in training"), and carry it in a visible place.	5.2
5	The health-care center (not the medical school) takes charge of providing me the student identification card for the clinical practice.	5.2
6	When the academic year is finished, I am obligated to give the student identification card back.	5.2
7	When assigning the rotary, the faculty informs me of what service is assigned to me. In addition, they also inform me of which doctor will be my responsible.	4.3
8	On the first day of each rotary, my assigned tutor already knew how many students we were and our names, our schedule and practice calendar, etc.	4.3
9	In preclinical years (mainly 1st and 2nd) we have used mannequins, simulated patients and/ or roleplays, in order to acquire skills for the "patient-student" relationship in subsequent clinical courses.	4.5
10*	I have got nervous on some occasion during a health care process in my clinical practice, and I have missed not having practiced before with a mannequin, simulated patient, using roleplays, etc.	4.5
11*	During my clinical practice, I have access to the patient's medical history and its modification.	7.4
12*	I do clinical interviews with patients and read their medical reports without the presence of my responsible doctor.	5.4
13	During my clinical practice, the doctor knocks at the door and greets the patients when entering their rooms.	4.1
14*	We have been present more than 3 students at the same time in some health-care process of a patient.	5.5
15*	I have sent a patient's medical history to my personal email, without expressly asking for permission.	4.8, 7.5
16*	I have used my personal mobile phone during a patient's care process for not related things with learning (WhatsApp, social networks, etc.)	4.7
17	My assigned doctor informs me about patients' rights and corrects me in case of not acting correctly on issues of personal treatment, ethics, privacy, dignity, etc.	4.1, 4.6
18	Before a patient's care process, my assigned doctor informs him/her (or his/her representative) of the presence of students and requests their verbal consent for the students to witness the clinical act.	5.2, 5.3
19	If the doctor considers appropriate for me to carry out some type of exploration or procedure, he again requests the verbal consent of the patient (or his representative).	5.3
20*	Some doctor told me private aspects about a patient without any clinical interest ("she is the mother of the mayor", "she is the sister of the hospital manager", "he likes to go to certain places", etc.).	8.2
21	The management of the health centers in which I carry out my training rotations (medical director, hospital manager, etc.) are responsible for explaining the ethical principles and basic rules of action in the patient care process.	4.6

22	During my clinical practice, the doctor introduces and identifies him/herself when entering the patients' rooms, as well as introduces me and identifies me as a student.	4.1, 5.2, 5.3
23*	During my clinical practice, I have access to patients' identification data, such as name, surname, age, address, etc. (I see it in the report, my medical doctor tells me, etc.), not only purely clinical data.	7.2, 7.4
24	The health-care centers in which I carry out my clinical practice have systems that allow students to read clinical reports about patients without being able to see their identification data (name, address, profession, etc.).	7.2

These questions were reversed when analyzed.

Supplementary Table S4. Questions 25-45 regarding students' opinion and articles related from the SSI/81/2017 Spanish Order.

Question	Statement	Articles
25*	Being a student and not a doctor, I am legally not subject to keep the medical secret.	8.2
26	If a patient gives his/her consent, the doctor is legally allowed to "break" the medical secret.	8.2
27*	If a patient dies, the medical secret disappears with him/her.	8.2
28*	During my clinical practice in the hospital, a patient wants me not to be in his care process because I am a student. I refuse, since it is a "university hospital".	5.3
29*	During your clinical rotation in Cardiology, a neighbor of yours gets hospitalized in your floor. When you get home, you tell your parents/friends about it.	8.1, 8.2
30	If a doctor diagnoses a patient with a Disease Of Public Health Significance or DOPHS (for example Cholera), the doctor is legally allowed to "break" the medical secret and report to health authorities.	8.2
31*	I take a photograph of a patient's admission report, which will be useful for preparing some assignment. I will not share it with anyone, I will simply store it in the phone's photo gallery.	4.8, 7.5
32*	During your clinical rotation in Psychiatry, a patient suspect for gender violence addresses you because he wants some medication to calm the headache. You are alone. You ignore it and don't tell your doctor.	4.1
33	If a doctor diagnoses a patient with a severe contagious disease, the doctor is legally allowed to "break" the medical secret and tell it to the patient's partner, even if the patient does not want, in order to avoid potential damage of partner's health.	8.2
34*	During my clinical practice, I see a patient with a Fournier's Gangrene. It is a pathology that is not seen every day, so I send a photo of the perineal necrosis to a WhatsApp group with class friends, without informing the patient, but without giving information about his identity.	4.8
35*	In case of a patient who a priori is going to spend a long stay in the plant (> 15 days), if the doctor has already requested the patient's verbal consent for me to be present at the clinical events and even perform physical examinations, it is not necessary to request his verbal consent again during the rest of his stay.	5.3
36*	During your emergency clinical rotation, a patient in the waiting room does not stop shouting and insulting the staff because he has been waiting his turn for quite some time. For this reason, you assist earlier other patients who came later than him.	4.1
37*	During your clinical rotation through the Internal Medicine plant, you perform a physical examination of a terminally ill patient. When finished, you go with your doctor to the hallway to continue passing floor. However, when you leave you realize that you have exposed the intimate parts of the patient and you tell the doctor. The patient has dementia and has no family / friends who could complain. The doctor tells you "it is not necessary; nobody comes to see him".	4.1
38	If a judge requests a doctor to testify at a trial, the doctor is legally allowed to "break" the medical secret regarding that patient.	8.2
39*	I upload to Instagram a photograph in which I am auscultating a patient. I do not expressly ask for permission. The patient has a characteristic tattoo on the sternum area (visible in the photograph), but his face cannot be seen.	4.8
40*	I upload to Instagram a photograph in which I am auscultating a patient. I do not expressly ask for permission, but its face cannot be seen and, in this case, there is no way to know its identity.	4.8

41*	During your surgical rotary in Traumatology, the surgeon photographs with her personal mobile phone the preoperative area of patients with large cosmetic defects. After the operation, she photographs the area again to make a comparison. In order to know which patient belongs each image, she labels its medical record number to the image.	4.8
42	In case of an under-18 patient, the doctor must request verbal consent (about my presence as a student in the care process) from the patient's legal representative.	5.3
43*	In the same previous case (under-18 patient), once verbal consent has been requested from the legal representative, the latter decides on his own, and has no obligation to listen to what the minor thinks about what has been reported.	5.3
44	In case of a patient with limited decision-making capacity, the doctor must request verbal consent (about my presence as a student in the care process) from the family/partner/legal representative of the patient.	5.3
45	In case of an incapacitated patient (with a judicial sentence), the doctor must request verbal consent (about my presence as a student in the care process) from the family/partner/legal representative of the patient.	5.3

These questions were reversed when analyzed.

Supplementary Table S5. Questions concerning situations related to students' experience concerning patient's right to privacy (question 1 to 24) and opinion regarding several assertions and their attitude when facing some situations related to the patient's privacy(questions 25 to 45)

Question number	Question	Score				
		0	0.25	0.50	0.75	1
1	During my clinical rotation, I am constantly supervised and accompanied by my doctor.	Never	Rarely	Sometimes	Quite often	Always
2	At the beginning of my rotations, I signed a confidentiality commitment? agreement provided by the health center where I carry out the practice.	No				Yes
3	During my clinical rotation, the doctor provided me with his/her username and password for the hospital's intranet.	Always	Quite often	Sometimes	Rarely	Never
4	When I am at the hospital, I wear the identification card (name and surname, photograph and "student in training"), and carry it in a visible place.	Never	Rarely	Sometimes	Quite often	Always
5	The health-care center (not the medical school) takes charge of providing me with a student identification card for the rotation.	No				Yes
6	When the academic year is finished, I am obligedto give my student identification card back.	No				Yes
7	When assigning the clinical rotations, the faculty informs me of what hospital service I am assigned to. In addition, they also inform me of which doctor will be responsible for my rotation.	Never	Rarely	Sometimes	Quite often	Always

8	On the first day of each rotation, my assigned tutor already knew how many students there would be, our names, our schedule and timetable, etc.	Never	Rarely	Sometimes	Quite often	Always
9	In preclinical years (mainly 1st and 2nd) we have used dummies, interactiveand/ or roleplays, in order to acquire skills for the "patient-student" relationship in subsequent clinical courses.	No				Yes
10	I have got nervous on some occasion during a health care procedure on my rotation, and I have missed not having practiced before with a dummies, interactive dummies, using roleplays, etc.	Yes				No
11	During my rotation, I have access to the patient's medical history and I could have modified it.	Always	Quite often	Sometimes	Rarely	Never
12	I carry out clinical interviews with patients and read their medical reports without the presence of my overseeing assigned doctor.	Always	Quite often	Sometimes	Rarely	Never
13	During my rotation, the doctor knocks on the door and greets the patients when entering their rooms.	Never	Rarely	Sometimes	Quite often	Always
14	More then 3 students have been present at the same time in some health-care procedures	Always	Quite often	Sometimes	Rarely	Never
15	I have sent a patient's medical history to my personal email, without	Yes				No

	expressly asking the patient for permission					
16	I have used my personal mobile phone during a patient's care procedure for things unrelated to learning (WhatsApp, social networks, etc.)	Always	Quite often	Sometimes	Rarely	Never
17	My assigned doctor informs me about patients' rights and corrects me in case of not acting correctly on issues of personal treatment, ethics, privacy, dignity, etc.	Never	Rarely	Sometimes	Quite often	Always
18	Before a patient's care procedure, my assigned doctor informs him/her (or his/her representative) of the presence of students and asks him/her for verbal consent for the students to witness the clinical act.	Never	Rarely	Sometimes	Quite often	Always
19	If the doctor considers it appropriate for me to carry out some type of exploration or procedure, he/she again asks for verbal consent of the patient (or his/her representative).	Never	Rarely	Sometimes	Quite often	Always
20	Some doctors told me private aspects about a patient without any clinical interest (e.g., "she is the mother of the mayor", "she is the sister of the hospital manager", "he likes to go to certain places", etc.).	Yes				No
21	The manager of the health centers in which I carry out my training rotations (medical director, hospital manager, etc.) are	Never	Rarely	Sometimes	Quite often	Always

	responsible for explaining the ethical principles and basic rules of action in the patient care process.					
22	During my rotations, the doctor introduces and identifies him/herself when entering the patients' rooms, as well as introduces me and identifies me as a student.	Never	Rarely	Sometimes	Quite often	Always
23	During my rotations, I have access to patients' identification data, such as name, surname, age, address, etc. (I see it in medical records or reports, my assigned doctor tells me, etc.), not only purely clinical data.	Always	Quite often	Sometimes	Rarely	Never
24	The health-care centers in which I carry out my rotations have systems that allow students to read clinical reports about patients without being able to see their identification data (name, address, profession, etc.).	No				Yes
25	Being a student and not a doctor, I am not legally obligedto keep medical secret.	Totally agree	Partially agree	Neither agree nor disagree	Partially disagree	Totally disagree
26	If a patient gives his/her consent, the doctor is legally allowed to “break” medical secret.	No				Yes
27	If a patient dies, medical secret disappears with him/her.	Yes				No
28	During my rotation in the hospital, a patient does not wans me to be present in	Totally agree	Partially agree	Neither agree nor disagree	Partially disagree	Totally disagree

	their care process because I am a student. I refuse, since it is a "university hospital".					
29	During your rotation in cardiology, a neighbor of yours gets hospitalized in your wards. When you get home, you tell your parents/friends about it.	Totally agree	Partially agree	Neither agree nor disagree	Partially disagree	Totally disagree
30	If a doctor diagnoses a patient with a disease of public health significance (for example cholera), the doctor is legally allowed to “break” medical secret and report to health authorities.	No				Yes
31	I take a photograph of a patient's admission report, which would be useful for preparing an assignment. I will not share it with anyone, I will simply store it in the phone's photo gallery.	Totally agree	Partially agree	Neither agree nor disagree	Partially disagree	Totally disagree
32	During your rotation in psychiatry, a patient suspected of domestic violence addresses you because he wants some medication to calm his headache. You are alone. You ignore it and don't tell your doctor.	Totally agree	Partially agree	Neither agree nor disagree	Partially disagree	Totally disagree
33	If a doctor diagnoses a patient with a severe contagious disease, the doctor is legally allowed to “break” medical secret and tell the patient's partner, even if the patient does not want it to be told, in order to avoid potential damage of partner's health.	No				Yes

34	During my rotation, I see a patient with a Fournier's Gangrene. This disease is not seen every day, so I send a WhatsApp group with class friends a photo of the perineal necrosis , without informing the patient, but without giving information about his identity.	Totally agree	Partially agree	Neither agree nor disagree	Partially disagree	Totally disagree
35	In case of a patient who is expected to stay in hospital more than 15 days, if the doctor has already asked the patient's for his verbal consent for me to be present at the clinical events and even perform physical examinations, it is not necessary to ask the patient againfor his verbal consent during the rest of his stay.	Totally agree	Partially agree	Neither agree nor disagree	Partially disagree	Totally disagree
36	During your emergency room rotation, a patient is continuously shouting and insulting the staff because he has been waiting to be seen for quite some time. For this reason, you attend to them before other patients who arrived later than him.	Totally agree	Partially agree	Neither agree nor disagree	Partially disagree	Totally disagree
37	During your rotation through the internal medicine wards, you perform a physical examination on a terminally ill patient. After finishing, you go with your doctor to the wards to continue visiting patients. However, when you leave the patient’s room you realize that you	Totally agree	Partially agree	Neither agree nor disagree	Partially disagree	Totally disagree

	have exposed the patient's genitalia and you tell the doctor. The patient has dementia and has no family / friends who could complain. The doctor tells you "it is not necessary; nobody comes to see him".					
38	If a judge requests a doctor to testify at a trial, the doctor is legally allowed to "break" medical secret regarding that patient.	No				Yes
39	I upload a photo to Instagram in which I am auscultating a patient. I do not expressly ask the patient for permission. The patient has a characteristic tattoo on the sternum area (visible in the photo), but his face cannot be seen.	Totally agree	Partially agree	Neither agree nor disagree	Partially disagree	Totally disagree
40	I upload a photo to Instagram in which I am auscultating a patient. I do not expressly ask the patient for permission, but her face cannot be seen, so there is no way to know her identity.	Totally agree	Partially agree	Neither agree nor disagree	Partially disagree	Totally disagree
41	During your rotation in traumatology, the surgeon photographs with her personal mobile phone anatomic areas of patients with large cosmetic defects. After the operation, she photographs the area again to make a comparison. In order to know which patient the image belongs to, she labels images with medical record numbers .	Totally agree	Partially agree	Neither agree nor disagree	Partially disagree	Totally disagree

42	In case of an under-18 patient, the doctor must ask the patient's legal representative for verbal consent about my presence as a student in the health care process.	Totally disagree	Partially disagree	Neither agree nor disagree	Partially agree	Totally agree
43	In the previous case (under-18 patient), once verbal consent has been asked for, the legal representative decides on his/her own, and has no obligation to listen to what the minor thinks about what has been reported.	Totally agree	Partially agree	Neither agree nor disagree	Partially disagree	Totally disagree
44	In case of a patient with limited decision-making capacity, the doctor must ask the family/partner/legal representative of the patient for verbal consent about my presence as a student in the care process.	Totally disagree	Partially disagree	Neither agree nor disagree	Partially agree	Totally agree
45	In case of an incapacitated patient (with a judicial sentence), the doctor must ask the family/partner/legal representative of the patient for verbal consent about my presence as a student in the care process.	Totally disagree	Partially disagree	Neither agree nor disagree	Partially agree	Totally agree
